# Mama Mach and Papa Mach: Parental Machiavellianism in Relation to Dyadic Coparenting and Adolescents’ Perception of Parental Behaviour

**DOI:** 10.5964/ejop.v14i1.1474

**Published:** 2018-03-12

**Authors:** András Láng

**Affiliations:** a Institute of Psychology, University of Pécs, Pécs, Hungary; Department of Psychology, Webster University Geneva, Geneva, Switzerland; Georgetown University, Washington, DC, USA

**Keywords:** Machiavellianism, perceived parenting behaviour, coparenting, disagreeableness, avoidant attachment

## Abstract

Parental personality is a main contributor to parenting outcomes. However, research on parental personality and parenting or coparenting behaviour is scarce. These few studies showed that disagreeableness and neuroticism are consequently related to negative parenting outcomes. Machiavellianism is an antagonistic and socially aversive personality trait. Machiavellianism has been linked to unfavourable outcomes in several different types of relationships (e.g., romantic relationships, workplace relationships). Using self-report measures, I investigated the association between parental Machiavellianism, adolescents’ perceptions of parenting behaviour, and parent reported coparenting in a sample of 98 families raising adolescents. According to the results, Machiavellianism was positively related to adolescents’ perceptions of rejecting and overprotective parenting behaviour in mothers. With regard to coparenting, fathers’ Machiavellianism had a negative effect both on their own and on their spouses’ reports of coparenting quality. Differences between fathers’ and mothers’ results are discussed with regard to their functions in the parent-child interaction and in the spousal dyad.

Machiavellianism has been in the focus of personality research since the beginning of the 1970s (Christie & Geis, 1970; Fehr, Samson, & Paulhus, 1992). Since then, this antagonistic and socially aversive trait has been known to make individuals less attractive mates (Rauthmann & Denissen, 2014) and less committed employees (Becker & O’Hair, 2007), but nothing has been revealed yet about the relationship between Machiavellianism and parenting qualities. At the same time, relatively much is known about the personality functioning and personality traits of Machiavellian individuals. Using these constructs to link Machiavellianism to parenting, I investigated the potential association between parents’ Machiavellianism, their adolescent children’s perception of their parenting behaviour, and parents’ reports of dyadic aspects of coparenting.

## Machiavellianism, Normal Personality Traits, and Personality Functioning

Machiavellianism is a personality trait characterized by interpersonal manipulation, cynical attitudes, and utilitarian morality (Christie & Geis, 1970). With regard to normal personality traits [i.e., traits in the Big Five (Costa & McCrae, 1992) and HEXACO (Ashton & Lee, 2007) models], Machiavellianism is consequently linked to low levels of agreeableness, conscientiousness, and honesty-humility (Lee & Ashton, 2005; Paulhus & Williams, 2002; Vernon, Villani, Vickers, & Harris, 2008). Some of the studies found Machiavellianism to be positively correlated with neuroticism as well (Jakobwitz & Egan, 2006; Vernon et al., 2008), whereas other studies reported no significant correlation between Machiavellianism and neuroticism (Lee & Ashton, 2005; Paulhus & Williams, 2002).

Considering the psychological maturity and differentiation of individuals with pronounced Machiavellian tendencies, McHoskey (2001) reported that Machiavellianism was associated with personality dysfunction in general and with the “dramatic” and “odd” clusters of personality disorders in specific. In this study by McHoskey (2001), Borderline Personality Disorder proved to be the best predictor of Machiavellianism among the DSM-IV (American Psychiatric Association, 1994) personality disorders. This result was further confirmed from a psychodynamic approach by Láng (2015) who found that Machiavellianism was predicted by Borderline Personality Organization as described by Kernberg (1985).

Findings from other areas of personality functioning further support the relative immaturity of Machiavellian individuals. Richardson and Boag (2016) found that Machiavellianism was related to an immature defensive strategy that was reflected in Machiavellian individuals’ frequent use of primitive defence mechanisms such as splitting, devaluation, and projective identification. According to Mudrack (1990), Machiavellianism is associated with an external locus of control, and interpersonal manipulation – a main characteristic of Machiavellian individuals – can represent an attempt to cope with a hostile, uncontrollable environment. Moreover, studies seem to agree that people with pronounced Machiavellian attitudes have deficits in empathizing and emotional intelligence (e.g., Ali, Amorim, & Chamorro-Premuzic, 2009; Baron-Cohen, 2011; Jones & Figueredo, 2013; Wastell & Booth, 2003).

## Parental Personality as a Determinant of Parenting

Based on available theory and clinical research, Belsky (1984) proposed a process model of parenting. According to this model, there are three interdependent general sources of influences on parenting: individual characteristics of parents (e.g., personality), individual characteristics of the child (e.g., temperament), and contextual factors (e.g., stress, social support). Belsky (1984) placed parental personality at the centre of this model, and he did it out of two reasons. On the one hand, personality affects parenting both directly and indirectly through influencing the context of parent-child relations (e.g., marital relations, social support). On the other hand, personality can be conceived of as a characteristic pattern of feelings, thoughts, and behaviours (Roberts, 2009). Thus, parents’ personality affects how parents feel, think and act in interaction with their children.

Compared with the aforementioned theoretical importance of parental personality for parenting quality, few studies investigated the association between Big Five personality traits and quality of parenting (e.g., Huver, Otten, de Vries, & Engels, 2010; Prinzie, Stams, Deković, Reijntjes, & Belsky, 2009; Vondra, Sysko, & Belsky, 2005). Since agreeableness, conscientiousness, and emotional stability (i.e., lack of neuroticism) are the three dimensions of the Big Five that are correlated with Machiavellianism, only parenting outcomes related to these three dimensions are reviewed here. In Huver et al.’s (2010) study with parents of adolescents and in the meta-analytic review by Prinzie et al. (2009), agreeableness was found to be positively related to warm, structured, and autonomy supportive parenting. Agreeableness showed negative correlations with parental insensitivity, negative control, and intrusive-overcontrolling behaviour – especially in mothers (Belsky, Crnic, & Woodworth, 1995).

In the previously mentioned meta-analytic review, Prinzie et al. (2009) reported modest but significant positive correlations between conscientiousness and parental warmth and positive control. Losoya, Callor, Rowe, and Hill Goldsmith (1997) found conscientiousness to be positively associated with supportive parenting and negatively related to negative parental control. With regard to neuroticism or emotional instability, Vondra et al. (2005) concluded that reviewed studies were consistent in reporting a negative relationship between neuroticism and competent parenting. However, Vondra et al. (2005) argued that parental incompetence could take the form of either uninvolved-neglecting or intrusive-overcontrolling parenting. Huver et al. (2010) found that emotional stability was negatively related to strict control. This result further supports that neuroticism might be related to intrusiveness and overcontrolling parenting as stated by Vondra et al. (2005).

In a previous edition of their chapter on personality and parenting, Vondra and Belsky (1993) reviewed studies that investigated the relationship between psychological differentiation and parenting. Psychological differentiation is an umbrella term for personality characteristics (e.g. having an internal locus of control, empathizing, and mentalizing) that enable the individual to function in a mature and integrated way. Vondra and Belsky (1993) argued that without these psychological resources for understanding and tolerating the stress related to parenting, parents were unlikely to become competent parents.

Recent research results are in accord with Vondra and Belsky’s (1993) aforementioned statement. Camberis, McMahon, Gibson, and Boivin (2016) found that hardiness – an indicator of psychological maturity – was related to more sensitive mothering in infancy. Johnson, McMahon, and Gibson (2014) found the same indicator of psychological maturity (i.e., hardiness) to be related to more favourable bedtime cognitions in mothers. In consequence, more mature mothers reported less sleep-related problems for their toddlers. Glatz, Cotter, and Buchanan (2017) found that parental self-efficacy predicted promotive (i.e., positive and involved) parenting in parents of adolescents. Parental self-efficacy can be considered as a form of internal locus of control which in turn is an indicator of psychological maturity.

## Parental Personality as a Determinant of Coparenting

Coparenting “refers to the ways that parents and/or parental figures relate to each other in the role of parents. Coparenting occurs when individuals have overlapping or shared responsibility for rearing particular children, and consists of the support and coordination (or lack of it) that parental figures exhibit in parenting” (Feinberg, 2003, p. 96). Whereas parenting styles refer to parental behaviours that occur in the child-parent interactions, coparenting describes parental interplay in raising children (Teubert & Pinquart, 2010). Several aspects of coparenting has been emphasized by different authors (Feinberg, 2003; Van Egeren, 2001; Van Egeren & Hawkins, 2004), but the essence of coparenting can be described by the following three components (Kolak & Volling, 2007). First, *cooperation* refers to the supportive and valuing attitude towards the other parental figure. Second, there may be *disagreement* between the parental figures about childrearing issues. Third, *triangulation* may occur. Triangulation refers to the parent’s efforts to form an alliance with the child against the other parental figure or to communicate with the other parent through the child.

Individual characteristics of parents – most dominantly personality traits and personality functioning – affect both coparenting and the parental relationship in general (Feinberg, 2003). Kolak and Volling (2007) applied the previously mentioned model of Belsky (1984) to describe the determinants of coparenting. According to this model, parent characteristics influence coparenting directly, but they also have an effect on coparenting through forming the parent’s relationship with the spouse, members of the broader family, and colleagues. Despite methodological and conceptual progress in the field, empirical research on the relationship between parental personality and coparenting is even scarcer than on the relationship between parental personality and parenting (McHale, Kuersten-Hogan, & Rao, 2004).

Weissman and Cohen (1985) described parents who are invested in their children, value the other parental figure’s childrearing efforts, respect the other parent’s judgements, and communicate with their partner as the best candidates for high quality coparenting. Laxman et al. (2013) found that fathers’ communion and negative emotionality were related to higher and lower coparenting quality, respectively. For mothers, they found that negative emotionality was associated with less undermining coparenting. Kolak and Volling (2007) found that positive expressiveness (i.e., being empathic, loving, and concerned) – especially that of fathers – was associated with more favourable coparenting outcomes. Stright and Bales (2003) found that parents’ personality adjustment – a constellation of high agreeableness, conscientiousness, extroversion, openness to experience, and low neuroticism – was positively related to supportive and negatively to unsupportive coparenting. To sum up these results in terms of the Big Five (Costa & McCrae, 1992), higher levels of coparenting seem to be associated with higher levels of agreeableness (investment, value, respect, communion, positive expressiveness) and lower levels of neuroticism (negative emotionality).

## Aim of the Study, Hypotheses

The aim of this study was to reveal how parental Machiavellianism was related to two aspects of parental functioning. On the one hand, I wanted to investigate the relationship between parents’ level of Machiavellianism and the way their adolescent offspring perceive them as parents. On the other hand, I wanted to reveal how parents’ ability to cooperate as parents (i.e., coparenting) was related to their level of Machiavellianism.

With regard to the relationship between Machiavellianism and adolescent reported parenting, the following hypothesis was formulated. Given the positive correlations between disagreeableness, emotional instability, lack of emotional intelligence / empathy, and Machiavellianism, parents with higher levels of Machiavellianism were expected to be perceived as less optimal parents by their adolescent offspring. I expected that less optimal parenting would be reflected in less parental emotional warmth, more parental rejection, and more parental overcontrolling (Hypothesis 1).

A further hypothesis (Hypothesis 2) was formulated with regard to the relationship between Machiavellianism and copareting. Given the fact, that Machiavellianism is a personality trait that is characterized by higher levels of disagreeableness, conscientiousness, and neuroticism, I expected that parents with higher levels of Machiavellianism will report lower quality of coparenting (actor effect) just like partners of individuals with higher levels of Machiavellianism (partner effect).

## Methods

### Participants and Procedure

Based on the expected strength of correlations (*r* = .35) and on conservative levels of power (.95) and significance (two-sided α = .05) we aimed to collect a sample of 100 families (Hulley, Cummings, Browner, Grady, & Newman, 2007). After excluding incomplete data, 98 cohabiting Hungarian families raising at least one 14-18 years old adolescent (target adolescent) participated in a study entitled ‘Personality traits of adolescents in a family system perspective’. This sample was deemed to guarantee adequate statistical power. Data from the same study was partially (overlapping variable: coparenting) used in Láng and Abell (2018) to investigate how interparental functioning related to adolescents’ level of Machiavellianism.

The relationship of parents lasted 21.59 years on average (*SD* = 3.83) and 96 pairs of parents were married. Mothers and fathers were 43.79 years old (*SD* = 3.57) and 46.54 years old (*SD* = 4.29) on average, respectively. The majority of parents had at least 12 years of education (55.1 and 66.3 percent for mothers and for fathers, respectively). Target adolescents (47 females) were all enrolled in formal education and were 16 years old (*SD* = 1.29) on average. Target adolescents had an average of 1.29 siblings (*SD* = .92).

Participants were recruited from the relational network of undergraduate psychology students. Students collected the data as partial fulfilment of the requirements for a developmental psychology research paper course. There was an inclusion criterion for the study. Only families composed of adolescents and two cohabiting biological parents were included in the study. Inclusion was independent of the amount of children. The inclusion criterion was introduced to obtain a relatively homogeneous sample with regard to family structure to ensure clarity for statistical analyses. Consenting family members participated voluntarily and anonymously in the study. Families received no reward in any form for the participation. The study received ethical approval from the United Ethical Review Committee for Research in Psychology (Ref. No.: 2016/063).

### Measures

#### Machiavellianism in Parents

Both mothers and fathers completed the Mach-IV Scale (Christie & Geis, 1970) as a measure of Machiavellian personality traits. In Mach-IV, participants are asked to report their agreement with 20 statements on a 7-point Likert-scale (1 = completely disagree; 7 = completely agree). Ten of these statements express Machiavellian attitudes (e.g., “Anyone who completely trusts anyone else is asking for trouble”), the other 10 statements express anti-Machiavellian attitudes (e.g., “One should take action only when sure it is morally right “). After reverse scoring anti-Machiavellian items, scores were summed and an additional 20 points were added to achieve 100 as the theoretical mid-point of the total score. This was done in line with the original formulation of Christie and Geis (1970). Mach-IV proved to be a reliable measure of Machiavellianism both for fathers and mothers (Table 1).

#### Adolescents’ Perception of Paternal and Maternal Parenting Behaviour

To measure their perception of paternal and maternal parenting behaviour, adolescents were asked to fill out the short Egna Minnen Beträffande Uppfostran (My memories of upbringing) Adolescent version (S-EMBU-A; Penelo, Viladrich, & Domènech, 2012). The S-EMBU-A is a 22-item self-report questionnaire that assesses perceived parental rearing style on the following three dimensions. (1) *Rejection* refers to criticizing and strict parental behaviour. This dimension is measured by 7 items (e.g., “My parents treat me in such a way that I feel ashamed”). To avoid loss of data from adolescents without siblings, the item referring to parental preference for siblings was omitted from latter analysis. (2) *Emotional Warmth* refers to loving and supporting parental behaviour. Emotional Warmth is measured by 6 items (e.g., “My parents praise me”). (3) *Overprotection* assesses overcontrolling and interfering parental behaviour with 9 items (e.g., “My parents want to decide how I should be dressed or how I should look”). Perceived frequency of the parental behaviours described in the items was reported on a 4-point Likert-scale (1=no, never; 4=yes, nearly always). Each item was evaluated separately for perceived paternal and maternal parenting behaviour. Scores were summed for each paternal and maternal dimension. All dimensions had good to excellent internal reliability (Table 1).

#### Parental Report of Dyadic Coparenting

Both mothers and fathers completed the parent form of Coparenting Inventory for Parents and Adolescents (CI-PA; Teubert & Pinquart, 2011). The inventory assesses the quality of coparenting on the following three subscales. (1) *Conflict* “is defined as the extent of parental arguments or fights over childrearing as well as the extent of undermining the other parent through criticism, disparagement, or blame” (Teubert & Pinquart, 2010, p. 287). (2) *Cooperation* “refers to the extent parents exchange information about their child, support and respect each other as parents, as well as communicate to the child a climate of mutual loyalty” (Teubert & Pinquart, 2010, p. 287). (3) *Triangulation* “includes coalition formation between a child and one parent, and involvement of the child in parental conflicts” (Teubert & Pinquart, 2010, p. 287). Each subscale is measured by 4 items (e.g., “Me and my partner agree on whether to fulfil the wishes and demands of our child or not (reversed)”, “Me and my partner reach shared decisions with regard to our child’s upbringing”, “Our child gets involved in the arguments between me and my partner” for conflict, cooperation, and triangulation respectively). Scores were summed to obtain a total score for each subscale. Conflict and triangulation were reverse scored. Thus, higher scores on each subscale indicate higher quality coparenting. Both mother- and father-reported subscales had Cronbach αs that showed excellent internal reliability (Table 3).

### Statistical Analyses

Means and standard deviations were computed for the measured variables. To indicate the internal reliability of scales, Cronbach αs were computed as well. Pearson’s correlations were used to test the linear relationships between pairs of variables. To further investigate the relationship between parental Machiavellianism and adolescents’ perception of their parents parenting behaviour, parental Machiavellianism was regressed on perceived parenting variables separately for mothers and fathers. Moreover, commonality analysis was used to reveal unique and common effects. “Unique effects identify how much variance is unique to an observed variable, and common effects identify how much variance is common to groups of variables” (Nimon, 2010, p. 10). The dyadic interdependence between parental Machiavellianism and reported coparetning behaviour was modelled by using IBM SPSS AMOS in accord with the Actor-Partner Interdependence Model (APIM; Kashy & Kenny, 1999; Kenny & Cook, 1999; Kenny, Kashy, & Cook, 2006). APIM is a promising solution to one of the challenges of relationship research, namely the nonindependence of dyadic data. Variable level data is available at osf.io/e4s2k.

## Results

Means, standard deviations and internal reliability indices (Cronbach αs) for the measured variables are presented in Tables 1 and 3. According to these results, all measures proved to be reliable in this study.

### Parental Machiavellianism and Perceived Parenting

First, the linear relationship between parents’ level of Machiavellianism and adolescents’ perception of their parents were analysed with Pearson’s correlations. Results of these analyses (Table 1) revealed that fathers with higher levels of Machiavellianism – as compared to fathers with lower levels of Machiavellianism – were perceived by adolescents as significantly more rejecting. The strength of this correlation was weak. Mothers with higher levels of Machiavellianism – as compared to mothers with lower levels of Machiavellianism –were perceived by adolescents as significantly more rejecting and more overprotective. The strength of these correlations was moderate.

Further, to control for the overlap between different dimensions of perceived parenting, parental Machiavellianism was regressed on the three dimensions of perceived parenting, using multiple linear regressions with enter method separately for fathers and mothers. Moreover, commonality analysis (Nimon, 2010) was used to show the unique and common association of perceived parenting variables with parental Machiavellianism. For fathers, the model including the three dimensions of perceived parenting did not significantly predict fathers’ level of self-reported Machiavellianism (*R*^2^ = .067; *F*(3,94) = 2.258, *p* = .087). Therefore, further analyses were omitted for fathers.

Results of multiple linear regression for mothers (Table 2) showed that the model including the three dimensions of perceived parenting accounted for 13.5 percent of the variance in mothers’ Machiavellianism (*F*(3,94) = 4.899, *p* = .003). After controlling for shared effects, only perceived overprotection remained a significant predictor of maternal Machiavellianism. Results of commonality analysis (Table 2) further revealed that the highest proportion (42.666 percent) of *R*^2^ was due to the common effect of maternal rejection and overprotection.

**Table 1:** Relationship Between Parents’ Self-Reported Machiavellianism and Adolescents’ Perception of Parenting Behaviour; Results of Pearson’s Correlations, Descriptive Statistics and Internal Reliability Indices

	*M*	*SD*	Cronbach α	2	3	4	5	6	7	8
1. Paternal Machiavellianism	98.306	15.819	.825	.233*	- .122	.184	.455***	.424***	- .358***	.326**
2. Adolescents’ perception of paternal rejection	8.612	2.559	.791		- .342**	.411***	.239*	.684***	- .240*	.280**
3. Adolescents’ perception of paternal emotional warmth	17.245	3.969	.852			.064	.002	- .265**	.731***	- .037
4. Adolescents’ perception of paternal overprotection	19.327	4.538	.741				.216*	.464***	.003	.787***
5. Maternal Machiavellianism	89.582	12.631	.726					.300**	- .120	.327**
6. Adolescents’ perception of maternal rejection	8.408	2.153	.731						- .435***	.471***
7. Adolescents’ perception of maternal emotional warmth	18.725	3.415	.816							- .064
8. Adolescents’ perception of maternal overprotection	21.031	4.956	.775							

*p < .05. **p < .01. ***p < .001.

**Table 2:** Mothers’ Machiavellianism Regressed on the Three Dimensions of Adolescents’ Perception of Parenting Behaviour; Results of Multiple Linear Regression and Commonality Analysis.

Variables	Commonality Coefficient	Percent of *R*^2^	β	*p*
Unique to R	.018	13.494	.172	.162
Unique to EW	.001	0.488	- .029	.789
Unique to OP	.045	33.268	.244	.029
Common to R and EW	.009	6.730		
Common to R and OP	.058	42.666		
Common to EW and OP	- .001	- 0.381		
Common to R, EW, and OP	.005	3.734		
Total	.135	100.0000		

*Note.* R = Rejection; EW = Emotional Warmth; OP = Overprotection.

### Mothers’ and Fathers’ Self-Reported Machiavellianism and Dyadic Coparenting

First, Pearson’s correlations were run to analyse the relationship between mothers’ and fathers’ self-reported Machiavellianism and dyadic coparenting (Table 3). Results revealed that mothers’ Machiavellianism was negatively correlated with all three dimensions of dyadic coparenting. This means that mothers with higher levels of Machiavellianism – as compared to mothers with less pronounced Machiavellian traits – reported more conflict with their spouses about child rearing issues, less cooperation in parenting, and more triangulation of the child. Moreover, two aspects of spouses’ report of dyadic coparenting quality was also correlated with mothers’ self-reported Machiavellianism. This means that men with spouses who are relatively more Machiavellian reported less cooperation in parenting and more triangulation of the child. With regard to fathers’ level of Machiavellianism, this was correlated with both self-reported and spouse reported conflict, cooperation, and triangulation. This means that fathers with higher levels of Machiavellianism reported lower quality coparenting as measured by all three subscales. Moreover, spouses of relatively more Machiavellian fathers reported lower quality coparenting on all three subscales as well. The strength of these correlations was week to moderate.

Using IBM SPSS AMOS and bootstrapping (*n* = 2000), modelling in accord with the APIM (Kenny et al., 2006) revealed both significant actor and significant partner effects (Table 4). According to the results fathers’ report of dyadic coparenting conflict, coparenting cooperation, and triangulation were only affected by their own level of Machiavellianism. The more Machiavellian the father the lower quality coparenting was reported by him on all three subscales. On the other hand, mother-reported coparenting was affected only by their partners’ level of Machiavellianism. It means that mothers’ perception of coparenting was less favourable on the subscales of coparenting cooperation and triangulation, if they had a coparent with more pronounced Machiavellian traits.

**Table 3:** Relationship Between Parents’ Self-Reported Machiavellianism and Dimensions of Coparenting; Results of Pearson’s Correlations, Descriptive Statistics and Internal Reliability Indices.

	*M*	*SD*	Cronbach α	2	3	4	5	6	7	8
1. Paternal Machiavellianism	98.306	15.819	.825	- .343**	- .446***	- .496***	.455***	- .254*	- .377***	- .411***
2. Fathers’ report of coparenting conflict	15.010	3.289	.865		.841***	.743***	- .153	.655***	.627***	.645***
3. Fathers’ report of coparenting cooperation	16.031	3.381	.868			.796***	- .296**	.616***	.686***	.695***
4. Fathers’ report of triangulation	15.031	3.762	.853				- .245*	.593***	.637***	.774***
5. Maternal Machiavellianism	89.582	12.631	.726					- .249*	- .260*	- .322**
6. Mothers’ report of coparenting conflict	14.837	3.232	.843						.775***	.763***
7. Mothers’ report of coparenting cooperation	15.857	3.485	.863							.729***
8. Mothers’ report of triangulation	15.245	3.567	.847							

*Note.* Copareting variables (conflict, cooperation, and triangulation) are scored so that higher scores indicate more cooperative copareting.

*p < .05. **p < .01. ***p < .001.

**Table 4:** Actor and Partner Effects of Machiavellianism on Dimensions of Coparenting; Results of Actor-Partner Interdependence Analyses.

Coparenting variable	Actor effects				Partner effects				*R*^2^		Correlation between errors of dependent variables	
	Fathers’ Machiavellianism to fathers’ coparenting		Mothers’ Machiavellianism to mothers’ coparenting		Fathers’ Machiavellianism to mothers’ coparenting		Mothers’ Machiavellianism to fathers’ coparenting					
	β	95% CI	β	95% CI	β	95% CI	β	95% CI	Father	Mother	*r*	95% CI
Conflict	-.345**	-.562, -.081	-.168	-.409, .068	-.178	-.435, .089	.005	-.216, .197	.118	.087	.633***	.461, -.761
Cooperation	-.393**	-.570, -.167	-.112	-.315, .089	-.326**	-.544, -.057	-.117	-.304, .061	.210	.152	.620***	.467, .751
Triangulation	-.485***	-.641, -.292	- .171	-.393, .039	-.333**	-.539, -.094	-.024	-.211, .157	.246	.192	.726***	.590, .828

*Note.* Correlation between fathers’ and mothers’ Machiavellianism is *r* = .455 (*p* < .05). Coparenting variables are scored so that higher scores indicate more cooperative coparenting.

*p < .05. **p < .01. ***p < .001.

## Discussion

This study was the first to investigate the relationship between the parental personality trait of Machiavellianism and two different aspects of parenting – parenting behaviour as perceived by adolescent offspring and self-reported coparenting behaviour. Results of the study partially confirmed my hypotheses. Results are discussed in the following two sections.

### Parental Machiavellianism and Perceived Parenting

As hypothesized (Hypothesis 1), both mothers’ and fathers’ level of Machiavellianism showed significant positive correlation with perceived rejecting parenting behaviour, but only mothers’ level of Machiavellianism was positively associated with perceived overprotection. Neither parents’ level of Machiavellianism was associated with perceived emotional warmth. Results of linear regression and commonality analysis showed that the relationship between dimensions of perceived parenting and parental Machiavellianism was significant only for mothers. Only overprotection emerged as a significant unique predictor accounting for 33.27 percent of maternal Machiavellianism’s *R*^2^. The greatest proportion of maternal Machiavellianism’s *R*^2^ (42.67 percent) was accounted for by the common effect of perceived maternal overprotection and rejection.

To explain the relationship between adolescents’ perception of parenting behaviour and maternal – but not paternal – Machiavellianism, the different roles of mothers and fathers in parenting should be considered. While mothers tend to be characterized by interpersonal concerns, instrumental concerns tend to describe fathers (Richards, Gitelson, Petersen, & Hurtig, 1991). This difference between mothers and fathers persists into adolescence – mothers continue to provide care and reassurance, whereas fathers foster autonomy and exploration (Duchesne & Ratelle, 2014). I speculate that the disagreeable nature of Machiavellianism (e.g., Jakobwitz & Egan, 2006; Paulhus & Williams, 2002) would interact rather with mothers’ relational than with fathers’ instrumental functions in socialization. This in turn would make maternal Machiavellianism – as compared to paternal Machiavellianism – correlate stronger with adolescents’ perception of parenting.

The main developmental task of adolescents in relation to their parents is separation and the development of autonomy. Parents can support this task with providing a secure base and fostering autonomy at the same time. Machiavellian mothers – lacking empathy, emotional intelligence (Ali et al., 2009; Andrew, Cooke, & Muncer, 2008; Wastell & Booth, 2003), and secure attachment (Brewer, Abell, & Lyons, 2013; Ináncsi, Láng, & Bereczkei, 2015; Jonason, Lyons, & Bethell, 2014; Láng & Birkás, 2015) – are speculated to be unable to sensitively attune to these needs of their adolescent offspring, which in turn makes them rejecting (i.e., failing to provide a secure base) and overprotective (i.e., failing to support autonomy).

Further, overprotection can be considered as an effort of Machiavellian mothers to exert control over their adolescent children. Although Machiavellian individuals rarely direct their manipulative actions towards family members (Barber, 1994), they perceive their environment and significant others to be unpredictable (Láng, 2015) and uncontrollable (Mudrack, 1990). In adolescence, children become more and more autonomous (Steinberg & Silverberg, 1986). This normative developmental process might be perceived by Machiavellian mothers as another instance of losing control. Therefore, overprotective parental behaviour might represent an effort of mothers with relatively high levels of Machiavellianism to maintain or regain control over their children.

### Parental Self-Reported Machiavellianism and Dyadic Coparenting

In partial accord with Hypothesis 2, both actor and partner effects were found between parents’ level of Machiavellianism and parents’ reports of coparenting. However, these effects were limited to the effect of fathers’ level of Machiavellianism. Both fathers and their spouses reported lower quality of coparenting if fathers had a relatively higher level of Machiavellianism.

For the selective effects of fathers’ Machiavellianism on both their own and their spouses’ evaluation of coparenting quality, the following explanations are offered. During the discussion of these results, two core aspects of Machiavellianism are highlighted: disagreeableness (e.g., Jakobwitz & Egan, 2006; Paulhus & Williams, 2002) and emotional detachment (Christie & Geis, 1970). Being disagreeable or antagonistic prevents individuals from cooperation and accommodation to others (Costa & McCrae, 1992). Moreover, Machiavellianism is associated with competing problem solving strategies as well (Mesko, Lang, Czibor, Szijjarto, & Bereczkei, 2014). Thus, fathers with relatively higher levels of Machiavellianism might be less willing to comply with their spouses, including parenting issues. They might rather stubbornly stick to their own ideas in parenting – leading to less coparenting cooperation and more coparenting conflict. More direct support for this explanation can be drawn from the studies of Laxman et al. (2013) and Kolak and Volling (2007) who found that the level of communion and positive expressivity (constructs similar to agreeableness) were positively related to high quality coparenting – especially for fathers. With regard to triangulation, alliance formation can be considered as a manipulative alternative to genuine relating for Machiavellian individuals (Jones & Paulhus, 2014). Thus, forcing the child to take side in parental conflicts could be a means for Machiavellian fathers to strengthen their positions in interparental rivalry.

The negative effect of fathers’ Machiavellianism on mothers’ reports of coparenting is speculated to be due to the following. Based on their relationship oriented socialization (e.g., Gilligan, 1982), women are especially sensitive to the presence of social support in their marital relationship. For example, Martos, Sallay, Nistor, and Józsa (2012) found in a Hungarian sample that their partner’s supportive coping was positively correlated with women’s marital satisfaction, but negatively with that of men. In a study by Bodenmann, Pihet, and Kayser (2006), both their own dyadic coping and that of their partner were significant predictors of martial satisfaction for women. At the same time, only their own dyadic coping was predictive of men’s marital satisfaction. Machiavellian individuals with their predominantly avoidant attachment (Brewer, Abell, & Lyons, 2013; Ináncsi, Láng, & Bereczkei, 2015; Jonason, Lyons, & Bethell, 2014; Láng & Birkás, 2015) are distressed by the closeness of others, which in turn prevents them to effectively provide social support (Richman, DeWall, & Wolff, 2015). Thus, Machiavellian fathers’ lack of the ability to provide support might lead to perceptions of poorer coparenting quality in their spouses. Moreover, avoidant attachment was found to be associated with an overestimation of negative emotions and hostile reactions during conflict and daily life (Overall, Fletcher, Simpson, & Fillo, 2015). Childrearing is a stressful task for most parents and coping with this stress requires constructive contributions (Vondra & Belsky, 1993). Fathers with higher levels of Machiavellianism – who are characterized by higher levels of avoidant attachment as well – are speculated to react in a hostile way to their spouses’ negative emotions in childrearing conflicts. Thus, these conflicts are rather escalated than resolved, which in turn leads to spouses’ reports of poorer coparenting quality.

### Limitations and Conclusions

Although this study was unique in investigating the relationship between parental Machiavellianism and parenting, some limitations of the study should be mentioned. First, as it is emphasized throughout the paper, the study relied solely on self-reports. No matter that data came from different informants, they are still at risk for perceptual bias. Second, the study is cross-sectional in design. Thus, establishing causal relationships between Machiavellianism and parenting variables is beyond the scope of this study. Third, families were selected so that only two-parent families were enrolled in this study. This was done in order to make the sample more homogeneous. However, this prevents the generalization of the results beyond families that consist of biological mother, biological father, and children. These limitations should be overcome by future research. This research should use observational data with regard to parenting and coparenting behaviours, and preferably a longitudinal design.

This study showed that maternal – rather than paternal – Machiavellianism was more predictive of adolescents’ perception of parenting behaviour, while fathers’ level of Machiavellianism was the unique predictor of intradyadic functioning in the parental dyad. These results might have two practical conclusions. First, Machiavellianism is only one expression of socially aversive and antagonistic personalities (e.g., Marcus & Zeigler-Hill, 2015; Paulhus & Williams, 2002). Based on the impressive results of this study, other dark personality traits should be included in the research of the association between parental personality and parenting or coparenting behaviour. This could be a fruitful investigation of personality characteristics that are situated at the borderline between normal and pathological (Jonason, Duineveld, & Middleton, 2015).

Second, results might have implications for interventions with adolescents and their families as well. Rejecting and overprotective parenting might prevent adolescents from completing their developmental tasks of separation and identity achievement (Steinberg & Silverberg, 1986), whereas marital discord – including a poorer quality of coparenting – is regarded by prominent parenting authors as the best familial predictor of child maladjustment (see Feinberg, 2003 for a review). Thus, both maternal and paternal Machiavellianism interferes with optimal adolescent development. The selective effect of mothers’ Machiavellianism on parenting and fathers’ Machiavellianism on coparenting suggest that the most optimal interventions should target the family as a unit of intervention. Decreasing Machiavellian attitude in parents could lead to fathers’ increased ability to cooperate with and provide support to mothers in coparenting issues and to mothers’ more sensitive approach to their adolescent offspring’s needs. These in turn could lead to more optimal adolescent development, helping youth to develop into autonomous and responsible adults.
